# From the right ventricle to the descending aorta: a Case Report of complex thrombotic events in a patient with nephrotic syndrome

**DOI:** 10.3389/fphar.2025.1514801

**Published:** 2025-01-29

**Authors:** Jiaojiao Chen, Longyin Zhu, Fen Zhou, Yuting Li, Tao Gu, Jing Xia

**Affiliations:** Department of Nephrology, The First Hospital Affiliated to Army Military Medical University (Southwest Hospital), Chongqing, China

**Keywords:** rivaroxaban, nephrotic syndrome, anticoagulation, hypercoagulability, thromboembolic event, case report

## Abstract

**Background:**

Nephrotic syndrome is characterized by significant proteinuria, hypoalbuminemia, edema, and hyperlipidemia. Patients frequently exhibit hypercoagulability on account of endothelial dysfunction and abnormal blood coagulation function, which significantly heighten the risk of thrombus formation. Although anticoagulant therapy is crucial for preventing thrombotic events, formulating individualized anticoagulation treatment plans for these patients remains a challenge in clinical practice due to variations in renal function and the distinct metabolic characteristics of anticoagulant medications.

**Case Introduction:**

This case report presents a 27-year-old male patient diagnosed with nephrotic syndrome. The etiological diagnosis was hepatitis B virus-associated glomerulonephritis, and the pathological diagnosis was mesangial proliferative glomerulonephritis. The patient had a relapse accompanied by bilateral pulmonary embolism and a right ventricular thrombus, and ultimately underwent thrombectomy. In the subsequent year, despite receiving adequate anticoagulation therapy with rivaroxaban, rare events of descending aortic mural thrombus and bilateral renal infarction occurred. The patient was treated with anticoagulation, endovascular exclusion with a descending aorta covered stent for the isolation of mural thrombus, and prolonged antiplatelet therapy. As of now, follow-up has shown no recurrence of thrombotic events.

**Conclusion:**

This case underscores the challenges associated with managing the hypercoagulability in patients with nephrotic syndrome and emphasizes the importance of personalized anticoagulation therapy. To improve patient outcomes, future research should focus on the selection of anticoagulant agents, dosage optimization, and monitoring strategies to enhance the safety and efficacy of anticoagulation treatment in this patient population.

## Introduction

Patients with nephrotic syndrome (NS) are at high risk for thrombotic events due to hypercoagulability (Kerlin et al., 2012). Although cardiac thrombosis is relatively rare, it can lead to severe consequences if it occurs. Research indicates that the hypercoagulability in these patients is associated with changes in blood rheology, a decrease in anticoagulant factors, and long-term immunosuppressive therapy (Wang et al., 2024). However, there remains a lack of in-depth understanding of the specific disease mechanisms and optimal treatment strategies for this population.

Here we report a case of a NS patient who developed thrombosis in the right ventricle and bilateral pulmonary arteries after relapse. Following thrombectomy of the right ventricle and bilateral pulmonary arteries, the patient’s condition improved significantly. However, despite adequate anticoagulant therapy with rivaroxaban, the patient still experienced thrombosis in other organs. This phenomenon suggests a potential risk of thrombus recurrence and raises discussions about the selection of anticoagulant medications. As a direct oral anticoagulant (DOAC), the efficacy and safety of rivaroxaban in this population merit further investigation. This report emphasizes the importance of individualized anticoagulation protocols in complex cases and the need for enhanced monitoring and attention to potential risks during treatment, thereby providing practical guidance for clinicians and promoting awareness and management of similar cases.

## Case presentation

**FIGURE 1:**
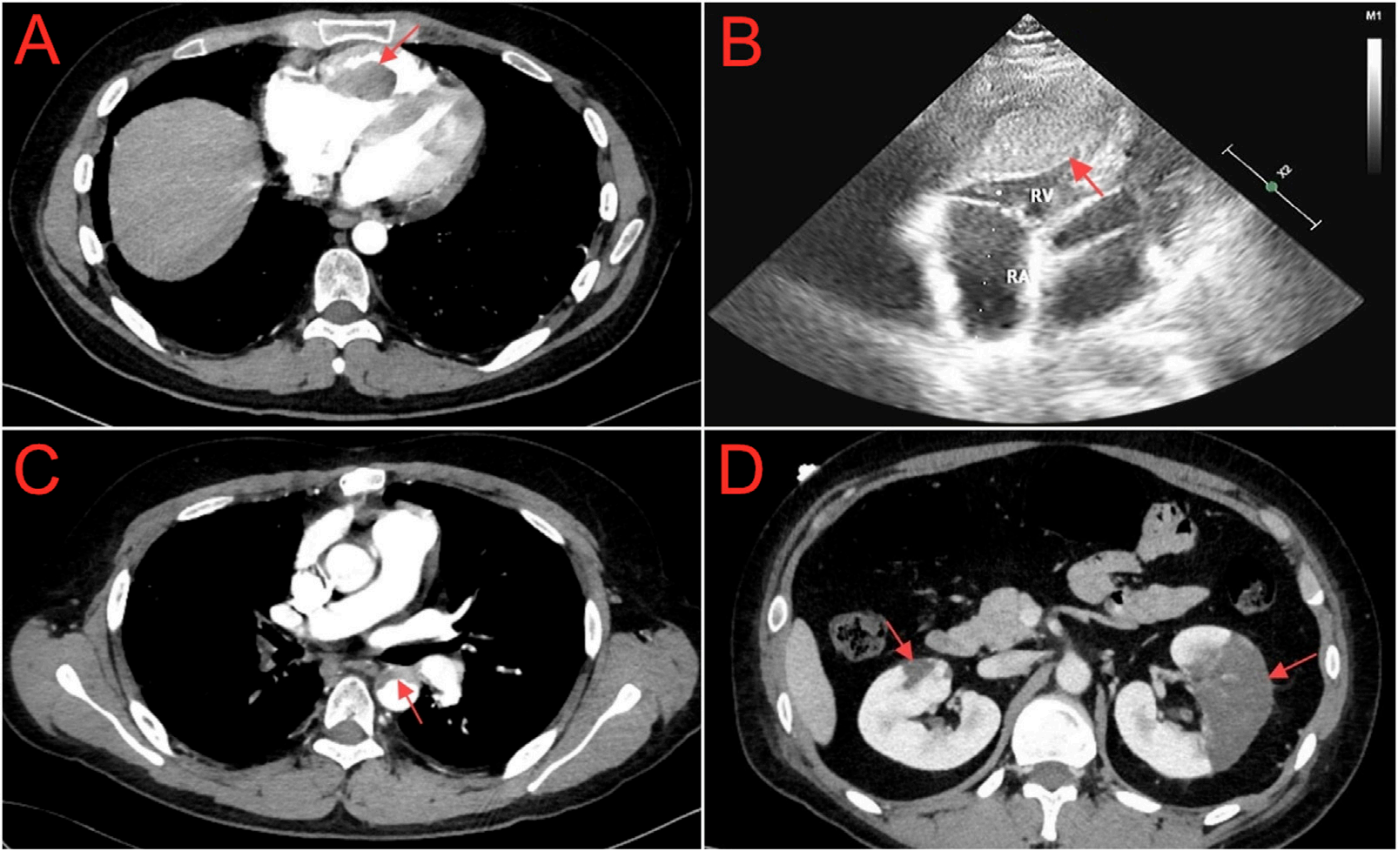
**(A)** Chest computed tomography angiography shows filling defect in the right ventricle (red arrow). **(B)** Echocardiogram shows a slightly hypoechoic area within the right ventricular cavity, with dimensions of approximately 32.4 × 20.9 × 61.9 mm (red arrow). **(C)** Abdominal computed tomography angiography shows a curved, non - enhancing, slightly low - density shadow within the lumen of the descending aorta (red arrow). **(D)** Abdominal computed tomography angiography shows irregular sheet - like low - density opacities in the renal parenchyma, notably more pronounced in the left kidney (red arrow).

On 24 October 2023, a 27-year-old Han Chinese male patient was admitted with complaints of left-sided flank and abdominal pain, accompanied by vomiting, oliguria, and fever. The patient had a history of chronic hepatitis B (CHB) and NS, with the cause of the NS diagnosed as hepatitis B virus-associated glomerulonephritis (HBV-GN), along with a pathological diagnosis of mesangial proliferative glomerulonephritis. The patient had no other comorbidities, and there was no significant family history. Over the past 12 years (from 2011 to 2023), he had multiple courses of glucocorticoids to control his condition but experienced frequent relapses after tapering the medication. One year before, the patient had a relapse of NS, complicated by bilateral pulmonary embolism and right ventricular thrombus (Figures 1A, B). Consequently, he underwent right ventricular thrombectomy and bilateral pulmonary artery thrombectomy on 24 October 2022. Postoperatively, he was prescribed rivaroxaban 15 mg q12 h for anticoagulation, along with methylprednisolone 24 mg qd as immunosuppression for NS, atorvastatin calcium 20 mg qn to regulate blood lipids, and tenofovir fumarate 25 mg qd for the treatment of CHB. A follow-up on 7 December 2022, showed a 24-h urine protein quantification of 33 mg, albumin at 43.2 g/L, D-dimer at 0.34 mg/L, total cholesterol at 6.41 mmol/L, and triglycerides at 2.34 mmol/L. Following medical advice, the dose of rivaroxaban was adjusted to 20 mg qd for continued anticoagulation. However, as the test for determining the rivaroxaban plasma level has not been initiated in our region, the corresponding determinations have not been carried out. Between 7 December 2022, and 20 July 2023, the patient attended regular follow-up visits. During this period, NS was well controlled, and the dose of oral methylprednisolone was gradually tapered. On 20 July 2023, follow-up indicated that urine tests showed negative protein, albumin at 45.4 g/L, total cholesterol at 4.64 mmol/L, and triglycerides at 2.4 mmol/L, confirming that the patient’s NS was in complete remission. Methylprednisolone was completely discontinued. After 20 July 2023, the patient continued anticoagulation therapy with rivaroxaban 20 mg qd, lipid-lowering therapy with atorvastatin calcium 20 mg qn, and tenofovir fumarate 25 mg qd for CHB. The patient attended outpatient follow-up every two months, and there was no recurrence of NS during this period.

On 24 October 2023, the patient was readmitted to our hospital with complaints of left flank and abdominal pain, accompanied by vomiting. Due to the patient’s medical history, the medications the patient was taking upon admission included: rivaroxaban 20 mg qd, tenofovir alafenamide fumarate 25 mg qd, and atorvastatin calcium 20 mg qn. Upon admission, physical examination revealed a body temperature of 37.7°C, a pulse of 87 beats per minute, a respiratory rate of 20 breaths per minute, and blood pressure of 119/82 mmHg. The patient presented with a facial grimace suggestive of pain and a 15-cm midline surgical scar from a previous procedure was observed. Tenderness was present in the left lower abdomen without rebound tenderness, along with percussion pain in the left kidney area. Mild pitting edema was observed in both lower limbs, while no other significant findings were noted. Laboratory investigations revealed a white blood cell count of 20.16 × 10^9^/L, with neutrophils constituting 79.5% and platelets at 130 × 10^9^/L. Renal function tests indicated a creatinine level of 79.80 μmol/L and an estimated glomerular filtration rate (eGFR) of 100.88 mL/min. Serum albumin was measured at 21.00 g/L. The lipid profile showed total cholesterol at 10.81 mmol/L, triglycerides at 5.22 mmol/L, high-density lipoprotein cholesterol (HDL-C) at 2.54 mmol/L, and low-density lipoprotein cholesterol (LDL-C) at 5.74 mmol/L. Urinalysis revealed 2+ protein and 1+ hematuria. Coagulation studies demonstrated a D-dimer level of 2.75 mg/L and fibrin degradation products at 11.6 mg/L. Hepatitis B virus-deoxyribonucleic acid was 4.32 × 10^4^ IU/mL. Abdominal computed tomography angiography (CTA) revealed a curved, non-enhancing, slightly low-density shadow within the lumen of the descending aorta (Figure 1C) and irregular, sheet-like low-density opacities in the renal parenchyma, which were notably more pronounced in the left kidney (Figure 1D). Based on the patient’s clinical presentation and auxiliary examination findings, a diagnosis of descending aorta thrombosis accompanied by bilateral partial renal infarction was established.

**FIGURE 2:**
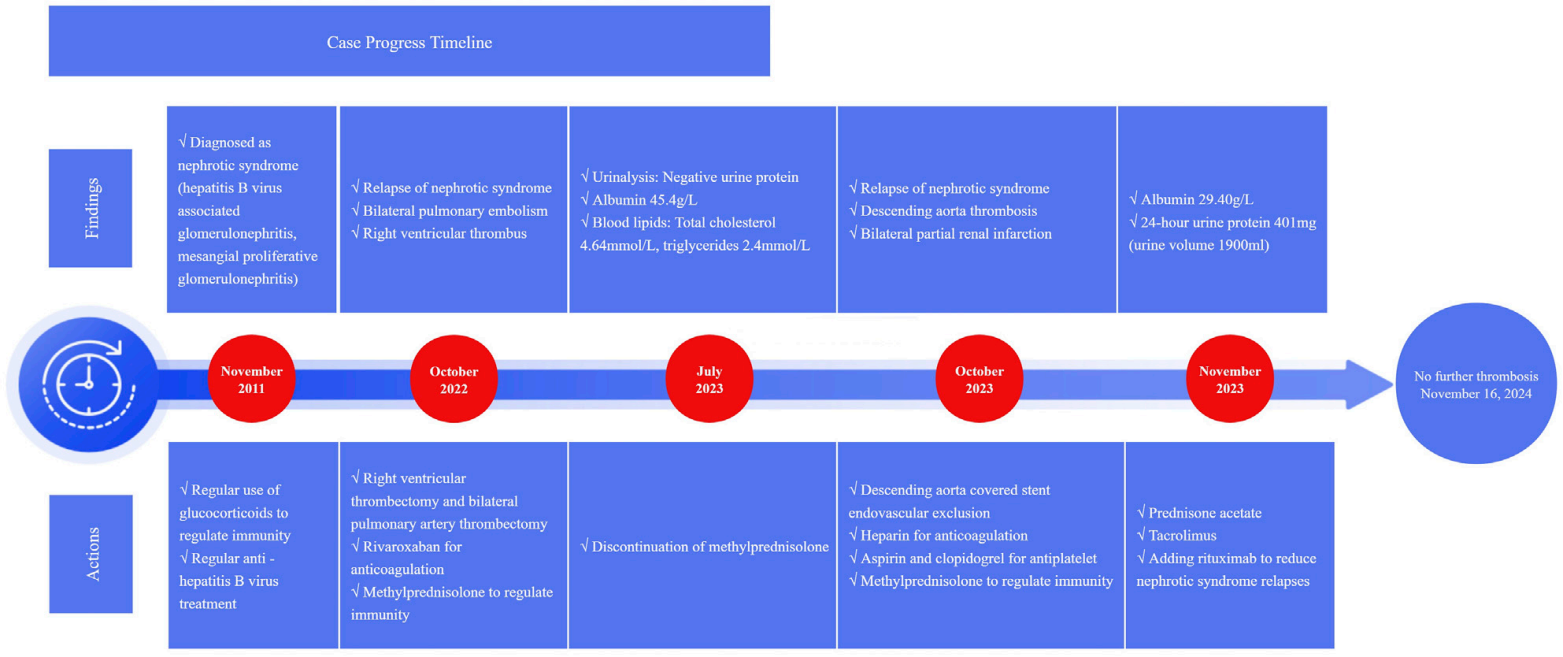
Case progress timeline.

Following admission, the patient was treated with enoxaparin sodium injection at 6000 IU q12 h for anticoagulation. On 25 October 2023, he underwent descending aorta covered stent endovascular exclusion for the isolation of mural thrombus. After the procedure, dalteparin sodium was initiated at a dose of 5000 IU q12 h for continued anticoagulation. On 27 October 2023, dalteparin sodium was discontinued, and the treatment regimen was adjusted to include aspirin 100 mg qd and clopidogrel 75 mg qd for antiplatelet therapy. Meanwhile, methylprednisolone was continued at 40 mg qd for immunosuppression, and tenofovir fumarate 25 mg qd was used for HBV. After treatment, the patient’s abdominal and flank pain symptoms were alleviated, and he no longer had a fever. The results of a follow-up examination conducted on 6 November 2023, indicated that the D-dimer level decreased to 0.97 mg/L, fibrin degradation products to 3.6 mg/L, and albumin increased to 29.40 g/L. Additionally, total cholesterol was measured at 6.2 mmol/L, triglycerides at 3.29 mmol/L, HDL-C at 1.4 mmol/L, and LDL-C at 3.85 mmol/L. Furthermore, the quantification of 24-h urine protein was 401 mg, with a total urine output of 1900 mL. Considering that NS has achieved a certain level of control following glucocorticoid therapy, the immunosuppressive regimen was adjusted to prednisolone acetate 30 mg qd and tacrolimus 1 mg q12 h. On November 7, rituximab 0.5 g was added to aid in alleviating NS and reducing recurrence (Due to the patient’s financial limitations, rituximab was not continued after this initial dose). The patient improved and was discharged on November 8, with follow-ups showing that NS was well controlled and no further episodes of thrombosis. The timeline illustrating progress of the case is shown in Figure 2 and Table 1.

**TABLE 1:** Table of case timeline progress.

Time node	Clinical signs	Laboratory results					Radiological investigations	Treatment and management strategies
		Alb(g/L)	D-dimer (mg/L)	TG (mmol/L)	Cr (µmol/L)	U-pro		
24 October 2022 (First Admission)	T: 36.5°C, P: 111 bpm, R: 25 breaths/min, BP: 138/104 mmHg. Mild lower - limb pitting edema	19.7	18.28	4.42	121.93	3+	Echocardiogram: Slightly hypoechoic area in right ventricular cavity (32.4 × 20.9 × 61.9 mm). Chest CTA: Low-density filling defects in left and right pulmonary arteries, massive filling defects in right ventricular cavity.	Right ventricular thrombectomy and Bilateral pulmonary artery thrombectomy Rivaroxaban 15 mg q12 h Methylprednisolone 24 mg qd Tenofovir fumarate 25 mg qd Atorvastatin calcium 20 mg qn
7 December 2022	Absence of positive signs	43.2	0.34	2.34	63.6	ND	ND	Adjustment of Rivaroxaban to 20 mg qd
20 July 2023	Absence of positive signs	45.4	0.27	2.4	61.3	(−)	ND	Gradual reduction of Methylprednisolone until drug withdrawal
24 October 2023 (Re - admission)	T: 37.7°C, P: 87 bpm, R: 20 breaths/min, BP: 119/82 mmHg. Left lower abdominal tenderness, left renal area percussion pain, and mild lower - limb pitting edema	21	2.75	5.22	79.8	2+	Abdominal CTA: 1. Curved, non-enhancing, slightly low-density shadow in descending aorta lumen; 2. Irregular sheet-like low-density opacities in renal parenchyma, more in left kidney.	Methylprednisolone 40 mg qd Tenofovir fumarate 25 mg qd Enoxaparin sodium injection 6000 IU q12 h 10/25 Descending aorta covered - stent endovascular exclusion, post - operation dalteparin sodium 5000 IU q12 h 10/27 Aspirin 100 mg qd + Clopidogrel 75 mg qd
6 November 2023	Absence of positive signs	29.4	0.97	3.29	ND	ND	ND	Prednisone acetate 30 mg qd + Tacrolimus 1 mg q12 h 11/7 add Rituximab 0.5 g
18 August 2024	Absence of positive signs	42.1	0.34	2.97	74.8	(−)	ND	Prednisone acetate and Tacrolimus are gradually tapered off until drug withdrawal.
16 November 2024	Absence of positive signs	44.2	0.28	2.11	74.8	(−)	ND	Discontinue Aspirin and Clopidogrel. Continue Tenofovir fumarate 25 mg qd for anti - hepatitis B virus treatment.

T, temperature; P, pulse; R, respiration; BP, blood pressure; bpm, beats per minute; Alb, Albumin; TG, triglyceride; Cr, Creatinine; U - Pro, Urine Protein; CTA, computed tomography angiography; q12 h, every 12 h; qd, once a day; qn, every night; ND, not detected.

## Discussion

Thrombosis is a multifactorial pathological process involving the interaction of several risk factors. The patient in this case has a history of CHB. Hepatitis B virus infection can trigger an inflammatory response, cause endothelial cell damage, and disrupt the balance of coagulation factors, thereby increasing the risk of thrombosis (Galli et al., 2014). However, the thrombotic mechanisms induced by hepatitis B virus infection are not the sole contributors. To investigate other potential causes, we conducted comprehensive autoantibody testing and excluded thrombotic risks associated with autoimmune diseases, including systemic lupus erythematosus, rheumatoid arthritis, antiphospholipid syndrome, inflammatory myopathy, Sjögren syndrome, systemic sclerosis, and vasculitis (Menichelli et al., 2023). Due to the limitations of the testing facilities at our medical institution, however, we were unable to perform thrombophilia screening (such as Protein C, Protein S, and Antithrombin) and genetic testing for hereditary thrombophilia, which precluded us from conducting a thorough evaluation of other potential thrombotic causes. Nonetheless, based on the patient’s clinical history and ancillary examination results, we conclude that the primary cause of thrombosis in this patient is closely associated with NS.

NS is a group of syndromes caused by various kidney diseases, characterized primarily by significant proteinuria (greater than 3.5 g/day), hypoproteinemia (serum albumin <30 g/L), edema, and hyperlipidemia (Tian et al., 2023). NS is often accompanied by multiple complications, including infections, thrombosis, acute kidney failure, cardiovascular complications, protein malnutrition, and disturbances in calcium-phosphate metabolism, among which thromboembolism is considered one of the most serious complications of NS (Orth and Ritz, 1998). Research indicates that approximately 27% of adults and 3% of children with NS experience thromboembolic events during the course of the disease (Waller et al., 2021), with an annual incidence of venous thrombosis (9.85%) slightly higher than that of arterial thrombosis (5.52%) (Zhao et al., 2016). Cardiac thrombosis is a relatively rare thromboembolic event among NS patients; however, it has a high mortality rate and is considered the most severe thromboembolic complication associated with NS (Aoyagi et al., 2002; Fukui et al., 2015; Hirano et al., 2001; Huang and Chau, 1995; Kioka et al., 1995; Mak et al., 1996; Malik et al., 1998; Raj et al., 2006). In this report, we describe a patient who underwent right ventricular thrombectomy and bilateral pulmonary artery thrombectomy one year ago and subsequently developed descending aortic and bilateral renal artery thrombosis despite sufficient anticoagulation treatment. This occurrence is exceedingly rare and warrants significant attention from clinicians. To date, no documented cases of this nature have been reported; therefore, we present this case for consideration.

In thromboembolic events associated with NS, renal venous thrombosis is the most prevalent, followed by pulmonary embolism and deep vein thrombosis (Gao et al., 2015). Contributing factors to thrombosis formation include venous stasis, endothelial injury within the venous system, and hypercoagulability, which collectively heighten the risk of thrombosis in individuals with NS. Currently, it is held that the mechanism underlying the predisposition to thrombosis in NS patients is primarily related to the disruption of the glomerular selective filtration barrier. This disruption leads to the leakage of a significant amount of large- and medium-molecular-weight proteins into the urine, including albumin and antithrombotic factors such as antithrombin III (AT-III), protein S, and tissue plasminogen activator (t-PA). The reduction in plasma albumin levels prompts the liver to increase compensatory synthesis of albumin. Concurrently, the synthesis of prothrombotic factors such as factor V, factor VIII, and fibrinogen also rises. This imbalance between prothrombotic and antithrombotic factors, along with platelet hyperfunction and decreased fibrinolytic system function, further exacerbates thrombosis formation (Glassock, 2007). In this case, the patient experienced a relapse of NS one year ago, which resulted in bilateral pulmonary embolism and a right ventricular thrombus. While pulmonary embolism is relatively common in thromboembolic events associated with NS, the occurrence of intracardiac thrombosis is notably rare. We hypothesize that the patient may have initially experienced asymptomatic bilateral pulmonary embolism, which obstructed pulmonary arterial flow, leading to increased right ventricular pressure and dysfunction. In response to this increased load, the hemodynamics of the right ventricle became sluggish, thereby elevating the risk of intracardiac thrombus formation. As the pressure burden on the right ventricle escalated, its contractile function declined, resulting in blood stasis that further exacerbated thrombus formation and established a vicious cycle. In addition, studies have shown that large and irregularly shaped right ventricular thrombus are more prone to dislodgement, thereby increasing the risk of pulmonary embolism (Cianciulli et al., 2008; Dhibar et al., 2016). For patients with NS, it is essential to conduct regular assessments of right ventricular function and pulmonary artery status to promptly identify the risk of thrombus formation and reduce the likelihood of intracardiac thrombosis.

Currently, there are no clear preventive anticoagulation guidelines for patients with NS in clinical practice, and there is limited relevant literature, lacking high-quality clinical research evidence. Membranous nephropathy is the type of NS that poses the highest risk of hypercoagulability and thrombosis, and thus, current anticoagulation evidence for NS mainly derives from membranous nephropathy (Gordon-Cappitelli and Choi, 2020). The 2021 Kidney Disease Improving Global Outcomes (KDIGO) guidelines recommend the use of heparin or warfarin as first-line prevention or treatment medications for thromboembolic events in membranous nephropathy (Radhakrishnan et al., 2024). However, heparin is primarily excreted through the kidneys, and its half-life is significantly prolonged in patients with renal insufficiency, which increases the risk of accumulation and subsequent bleeding. Warfarin has a narrow therapeutic window, interacts with numerous drugs and foods, and requires regular monitoring of coagulation parameters, posing certain limitations in its use (Barbano et al., 2013). The patient in this case underwent right ventricular thrombectomy and bilateral pulmonary artery thrombectomy. According to the Chinese Consensus on Antithrombotic Therapy for Acute Thrombotic Diseases (Ma et al., 2019), among patients with pulmonary embolism who do not have concurrent malignant tumors and are suitable for DOAC, dabigatran or rivaroxaban is recommended for long-term anticoagulation. Additionally, the results of a large-scale clinical trial indicate that rivaroxaban is as effective as traditional standard regimens in treating venous thromboembolism, while exhibiting a lower incidence of major bleeding (Buller et al., 2012). Studies have confirmed that patients with NS receiving DOAC experience a reduced risk of venous and arterial thrombotic events without an increased risk of bleeding (Van Meerhaeghe et al., 2022). Based on the above evidence, the patient complied with the doctor’s recommendation and took rivaroxaban for long-term anticoagulation after the operation. Unfortunately, one year later, the patient developed descending aortic thrombosis and bilateral renal infarctions.

Rivaroxaban is a potent selective oral direct factor Xa inhibitor. Its plasma protein-binding rate is approximately 92%–95%, primarily due to binding to serum albumin (Steffel et al., 2021). Rivaroxaban features rapid absorption, quick onset of action, and high bioavailability. Furthermore, it offers the advantages of not requiring routine monitoring of coagulation indices, a relatively fixed dosage that does not necessitate frequent adjustment, and fewer interactions with other drugs and food (Cohen and Bauersachs, 2019; Douketis et al., 2012). Numerous studies have shown that the anticoagulant effect of rivaroxaban is non-inferior to that of traditional anticoagulants and is associated with a lower incidence of adverse reactions (Van Meerhaeghe et al., 2022; Ge et al., 2020; Lü et al., 2020). However, in this case, the patient with NS developed thrombosis despite adequate treatment with rivaroxaban. This may be related to several factors. In patients with NS, drugs with a high protein-binding rate are influenced by urinary protein loss. This can lead to increased urinary excretion of the drug, thereby reducing the plasma concentration of the active drug and ultimately resulting in treatment failure. Additionally, significant albumin loss in urine may stimulate the liver to increase synthesis of various procoagulant proteins, including factor Xa (Kerlin et al., 2012). Given that rivaroxaban is a direct factor Xa inhibitor, its anticoagulant effect could be compromised by elevated factor Xa levels, leading to treatment failure. Furthermore, a study has indicated that the free fraction of rivaroxaban in NS patients is subject to increased metabolism and excretion, which accelerates the release of the bound portion and increases plasma clearance rates (Reynolds et al., 2019). Consequently, the plasma concentration of rivaroxaban may fail to reach therapeutic levels, resulting in treatment failure. Although studies have confirmed the effectiveness of rivaroxaban for prophylactic anticoagulation in NS patients, its application may differ from that in patients with other conditions. To improve the care of these patients, future efforts should focus on multiple aspects. In monitoring, beyond routine coagulation indicators, more effective monitoring methods should be explored for patients using specific anticoagulants, such as rivaroxaban, including measuring drug plasma concentrations or relevant coagulation factor levels to adjust treatment plans in a timely manner. Regarding drug selection, large-scale clinical trials comparing the efficacy and safety of different anticoagulants at different stages of the disease are needed to provide evidence for precise clinical medication. At the same time, strengthening patient education, improving adherence to treatment, and conducting regular follow-up evaluations are essential for timely identification of potential complications and adjustment of treatment strategies to improve patient outcomes.

## Conclusion

In summary, this case report emphasizes the importance of implementing individualized anticoagulation therapy in patients with NS. These patients are at an elevated risk of thrombosis due to prolonged use of immunosuppressants and the presence of hypercoagulability. Despite the standard anticoagulation regimen being employed, the patient in this case still developed thrombosis, indicating that we must closely monitor and adjust the anticoagulation plan throughout the course of the disease. In clinical practice, physicians should tailor anticoagulation and antiplatelet treatment approaches based on the patient’s renal function, comorbidities, and bleeding risk to minimize thrombosis incidence and enhance prognosis. Moreover, further research into the efficacy and safety of various anticoagulants in patients with NS is essential for optimizing anticoagulation therapy.

## Data Availability

The original contributions presented in the study are included in the article/supplementary material, further inquiries can be directed to the corresponding author.
